# Towards next‐generation TIL therapy: TILs enriched in neoepitope‐specific T cells

**DOI:** 10.1002/ctm2.1174

**Published:** 2023-01-11

**Authors:** Marion Arnaud, George Coukos, Alexandre Harari

**Affiliations:** ^1^ Ludwig Institute for Cancer Research, Lausanne Branch, Department of Oncology, University of Lausanne (UNIL) and Lausanne University Hospital (CHUV) and Center for Cell Therapy CHUV‐Ludwig Institute Lausanne Switzerland

**Keywords:** immunology, immunotherapy, T cells

1

The relationship between neoantigen recognition by T cells and the clinical success of cancer immunotherapy is beyond doubt.^1^, ^2^ Personalized neoantigen‐based cancer vaccines and neoantigen‐specific T‐cell therapy represent promising cancer treatments.^3^ Challenges yet remain to unleash the full potential of mutanome‐based cancer immunotherapy. Among them, the identification and validation of clinically‐relevant neoantigens require a complex, individualized and costly approach which only yields a limited number of neoepitope per patient.^4^, ^5^

We recently reported a novel method that enables the sensitive detection of tumour neoantigens in highly enriched in vitro‐expanded tumour‐infiltrating lymphocytes (TILs).^6^ This strategy, dubbed NeoScreen, is based on the early exposure of patients’ tumours to private sets of selected neoantigens loaded on competent autologous B cells. NeoScreen demonstrated a considerable enrichment in neoepitope‐specific T cells by several orders of magnitude and increased by threefold the breadth of tumour antigens found with conventional methods, thus enabling the sensitive identification of rare patient‐specific neoantigens. By increasing the frequency of existing tumour‐specific T‐cell receptor (TCR) clonotypes but also by recruiting additional clonotypes that can be newly detected with markedly enhanced sensitivity, NeoScreen allows the generation of expanded TILs highly enriched in neoepitope‐specific T cells.

Of note, in the TIL adoptive cell transfer therapy (ACT) field, both the breadth and magnitude of neoantigen‐specific T‐cell responses, together with tumour mutational burden, correlate with clinical outcome.^7^, ^8^ Collectively, this suggests that NeoScreen‐enriched TIL products represent attractive candidates for TIL ACT therapy. This perspective, however, raises important questions.

**FIGURE 1:**
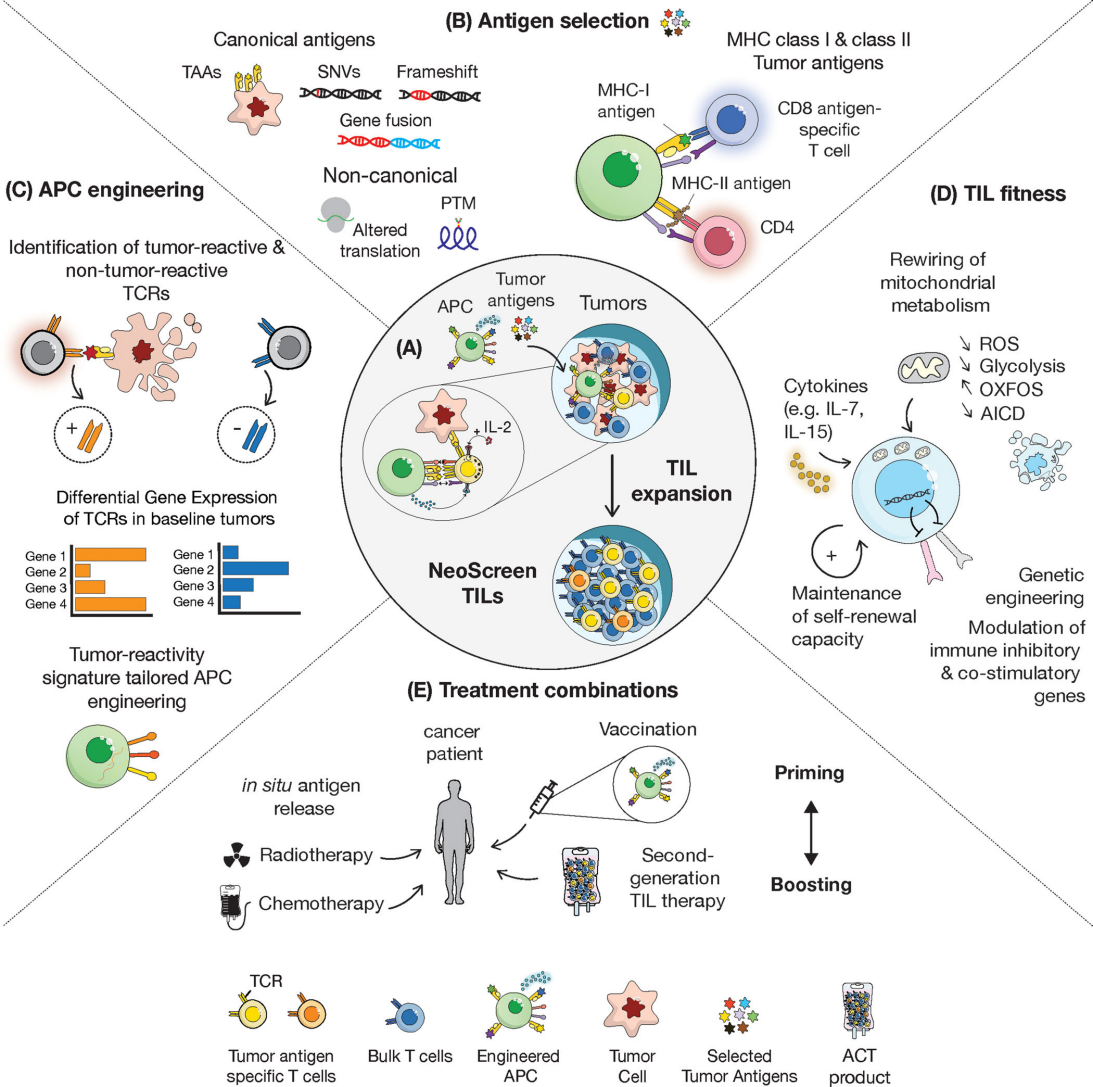
Next‐generation tumour‐infiltrating lymphocyte (TIL) therapy. Overview of strategies to improve TIL therapy **(A) NeoScreen principle**. **(B) Antigen selection**. Various antigens may be selected for TIL enrichment, including canonical: tumour‐associated antigens, TAAs, single‐nucleotide variants, SNVs, frameshifts or gene fusions; and non‐canonical antigens, derived for instance by altered translation or post‐translational modifications (PTMs). MHC class I‐ and class II‐restricted tumour antigens may be selected in order to ensure the stimulation of both CD8 and CD4 antigen‐specific TILs. **(C) APC engineering**. Based on the single‐cell RNA‐Seq profile of tumour‐reactive and non‐tumour‐reactive clonotypes, combined to single‐cell TCRseq, differential gene expression analysis can be performed in order to identify signatures of tumour reactivity. The defined signatures can then be used for tailored B cell engineering. **(D) TIL fitness**. The TILs’ metabolism may be improved by delaying T cell differentiation using medium supplementation with given cytokines, by maintaining the self‐renewal capacity of T cells or by genetic engineering (down‐regulation of immune‐inhibitory genes or up‐regulation of co‐stimulatory genes). **(E) Treatment combinations**. Next‐generation adoptive cell transfer therapy (ACT) may be first potentiated by releasing antigens in situ using standard‐of‐care cancer treatments (radiotherapy, chemotherapy), then anti‐tumoural immunity could be primed by vaccination prior to ACT (prime‐boost approach).

One key component is the nature of tumour‐rejection antigens to be selected and how to prioritize them (Figure 1), given that both neoepitope‐ and tumour‐associated antigen‐specific TILs were successfully enriched with NeoScreen. There is currently an armamentarium of computational tools to predict patient‐specific tumour antigens‐based, among others, on the affinity and stability of binding to cognate HLA alleles, immunogenicity, clonality, agretopicity or distance to self.^9^, ^10^ Furthermore, the spectrum of canonical and non‐canonical antigens is also expanding, including, among others, canonical neoantigens derived from indels or gene fusions and non‐canonical antigens such as post‐translational modifications or long noncoding RNAs.^5^, ^11^, ^12^

When selecting antigen candidates for TIL enrichment, and in particular with the perspective of exploring non‐canonical antigens, it becomes critically important to develop tools to assess antigens cross‐reactivity, that is the off‐target toxicity of enhanced cell products. Computational tools such as those estimating the dissimilarity of candidate antigens to the human proteome or molecular modelling tools predicting the likelihood of a given TCR to interact with distinct antigens are promising but are still in their infancy.^13^

In the NeoScreen study, only CD8 T cell responses were reported, although neoepitope‐specific CD4 TILs may also be enriched. CD4 help was shown to be key to promote and support anti‐tumour CD8 responses during successful immunotherapy.^14^ Moreover, the direct clinical relevance of neoantigen‐specific CD4 T cells is now beyond doubt^15^ and computational tools to predict MHC class II‐restricted neoantigens are improving.^16^ It will therefore be critical to achieve the generation of TIL products enriched in both MHC class I‐ and MHC class II‐restricted neoantigen reactivity for ACT (Figure 1).

Of interest, B cell engineering may be further improved to potentiate the stimulation of tumour antigen‐specific clonotypes. Via TCR discovery engines such as NeoScreen and also via alternative strategies, including the screening of the most frequent clonotypes in patients’ baseline tumours, ^17^ the profile of tumour‐reactive clonotypes is getting more and more granular. Investigators can thus capitalize on the latter dataset to investigate thoroughly the transcriptomic profile of tumour‐reactive TCRs in situ at the single‐cell resolution.^18^ The state and profile of tumour‐reactive TCRs may then be harnessed to tailor B cell engineering in NeoScreen (Figure 1) and thus to potentiate the targeted expansion of relevant tumour‐rejection TILs.

In the context of ACT, it is well established that cell state in infusion products is of paramount importance.^19^ Most of the tumour‐reactive TILs, which are the key players in ACT, fall into the exhausted compartment, due to repetitive TCR stimulation in vivo and ex vivo during cell expansion.^20^ Terminally differentiated TILs display specific ‘dysfunctional’ phenotypic and metabolic features and are characterized by the loss of the ability to self‐renew and by a poor persistence in vivo. Yet, recent evidence suggest that a small fraction of tumour‐specific TILs can retain stem‐like properties ex vivo and their presence in TIL products was restricted to melanoma patients responding to ACT therapy.^19^ Given the selective stimulation of neoantigen‐specific T cells with NeoScreen, methods to optimize TILs’ stemness will be fundamental to enhance the clinical benefit of TIL therapy. Among the many possible metabolic interventions (Figure 1) are the addition of memory cytokines during T cell expansion,^21^ the inhibition of signalling pathways leading to T cell effector differentiation^22^ or the prevention of T cell activation‐induced cell death by supplementation with anti‐oxidants.^23^ In addition, TILs can be re‐programmed towards memory and stemness by direct genetic engineering.^22^, ^24^ Importantly, the timing and combinations of manufacturing‐compatible interventions, either pharmacological or genetic, remain to be investigated.

Next‐generation ACT could be further improved using treatment combinations. Of interest, TIL therapy may be combined with standard‐of‐care cancer treatments such as chemotherapy and radiotherapy which can release tumour antigens, thus recruiting cellular immunity.^25^ Alternatively, a prime‐boost strategy including prior vaccination could first allow the mobilization of tumour‐specific T cells and then TIL therapy could synergistically enhance anti‐tumour immunity.^3^, ^25^ Moreover, vaccination may be used as a boost following ACT in order to promote efficient re‐stimulation and infiltration of adoptively transferred TILs in vivo.^26^ Overall, the establishment of relevant combination strategies should foster significant improvement in clinical benefit.
